# Towards individualized cortical thickness assessment for clinical routine

**DOI:** 10.1186/s12967-020-02317-9

**Published:** 2020-04-03

**Authors:** Marlene Tahedl

**Affiliations:** 1 Department of Psychiatry and Psychotherapy, University of Regensburg, Regensburg, Germany; 2 Institute for Experimental Psychology, University of Regensburg, Regensburg, Germany

**Keywords:** Cortical thickness, Neuroimaging, Magnetic resonance imaging (MRI), Individual diagnosis, Atrophy, Neurological assessment

## Abstract

**Background:**

Cortical thickness measures the width of gray matter of the human cortex. It can be calculated from T1-weighted magnetic resonance images (MRI). In group studies, this measure has been shown to correlate with the diagnosis/prognosis of a number of neurologic and psychiatric conditions, but has not been widely adapted for clinical routine. One of the reasons for this might be that there is no reference system which allows to rate individual cortical thickness data with respect to a control population.

**Methods:**

To address this problem, this study compared different methods to assess statistical significance of cortical thinning, i.e. atrophy. All compared methods were nonparametric and encompassed rating an individual subject’s data set with respect to a control data population. Null distributions were calculated using data from the Human Connectome Project (HCP, n = 1000), and an additional HCP data set (n = 113) was used to calculate sensitivity and specificity to compare the different methods, whereas atrophy was simulated for sensitivity assessment. Validation measures were calculated for the entire cortex (“cumulative”) and distinct brain regions (“regional”) where possible.

**Results:**

The approach yielding the highest combination of specificity and sensitivity implemented generating null distributions for anatomically distinct brain regions, based on the most extreme values observed in the population. With that method, while regional variations were observed, cumulative specificity of 98.9% and cumulative sensitivity at 80% was achieved for simulated atrophy of 23%.

**Conclusions:**

This study shows that validated rating of individual cortical thickness measures is possible, which can help clinicians in their daily routine to discover signs of atrophy before they become visually apparent on an unprocessed MRI. Furthermore, given different pathologies present with distinct atrophy patterns, the regional validation proposed here allows to detect distinct patterns of atrophy, which can further enhance differential diagnosis/prognosis.

## Background

Using magnetic resonance imaging (MRI), images with high-tissue contrast [1] of the brain can be acquired without making use of radioactive contamination of patients. Beyond clinical applications, MRI has been widely used for neuroscientific studies. Constantly, methods are being developed which allow to quantify biologic characteristics of the central nervous system and its constituents more and more differentiated, encompassing blood flow, nerve fiber myelination and properties of the cortex or “gray matter” (GM). The GM is the location of the neuron bodies, whereas the extent of cortical thickness seems to be related to synaptic density, synaptic pruning and intracranial myelination [2–5], rather than the number of neurons [5, 6]. A T1-weighted MRI of the brain is sufficient to compute cortical thickness in an automated procedure and can be further optimized with an additional T2-weighted image [7, 8]. Common algorithms to calculate cortical thickness are publicly available, e.g. under the open-source software package FreeSurfer [9].

Cortical thickness has been subject to a wide range of studies, and cortical thinning (i.e. atrophy) has been associated with diagnosis and progression of a number of neurologic conditions, such as Alzheimer’s Disease [10], Parkinson’s Disease [11] and Multiple Sclerosis [12] as well as psychiatric conditions, such as depression [13] and schizophrenia [14]. Interestingly, such pathological conditions present with different patterns of cortical thinning and are modified by age and genetic components [15, 16]. These specific aspects make cortical thickness a good candidate as a biomarker for differential diagnosis/prognosis. However assessing cortical thickness is rarely incorporated in clinical practice. One of the reasons for this might be the lack of a standardized system, based on which an individual’s cortical thickness data can be rated. To pass this limit, the present study aimed to develop a method to rate an individual’s cortical thickness data with respect to a control population which detects cortical atrophy with high sensitivity and specificity. To allow detecting distinct patterns of cortical atrophy, the tested methods allow the evaluation of separate brain regions. Such a standardized procedure can help clinicians detect early signs of distinct atrophy patterns and monitor their progression.

## Methods

### Subjects

In order to rate an individual’s data with respect to a control population, a large number of standardized data from a representative population sample is required. The Human Connectome Project (HCP) provides such a resource [17–19]. For this study, data from the HCP’s 1200 Subject Release was used. In total, structural data (T1- and T2-weighted sequences) from 1113 subjects was available at the time of this study (507 males, aged between 22 and 40). Of the 1113 subjects, 1000 were randomly selected for generating null distributions of cortical thickness, the rest was spared for subsequent validation (see below).

### Data acquisition and preprocessing

The HCP data was acquired on a 3 Tesla Connectome Scanner. Two different types of structural sessions were acquired, encompassing a T1-weighted MPRAGE (repetition time (TR) = 2400 ms, echo time (TE) = 2.14 ms, inversion time = 1000 ms, flip angle (FA) = 8°, field of view (FOV) = 224 × 224, voxel resolution (VR) = 0.7 mm^3^, bandwidth (BW) = 210 Hz/Px, iPAT factor 2, total acquisition time 7 min 40 s) and a T2-weighted SPACE (TR = 3200 ms, TE = 565 ms, FA variable, FOV = 224 × 224, VR = 0.7 mm^3^, BW = 744 Hz/Px, iPAT factor 2, total acquisition time 8 min 24 s). The full imaging protocols can be found online at http://protocols.humanconnectome.org/HCP/3T/imaging-protocols.html. All study procedures of the HCP protocol were approved by the Institutional Review Board at the Washington University in St. Louis.

The HCP offers data which was preprocessed with standardized and validated procedures. The main preprocessing steps encompassed gradient distortion correction, brain extraction, nonlinear registration, surface registration, and registration onto high-resolution (164 k mesh) and low-resolution (32 k mesh) templates; more details on the exact preprocessing pipeline can be found in [9, 20–22]. The image format of the mesh images is in CIFTI format (Connectivity Informatics Technology Initiative), a file format which combines surface-based cortical data with volumetric-based subcortical/cerebellar data, which was found to enhance alignment to the geometry of the cortex as well as statistical power [23]. The HCP’s minimally preprocessed data include cortical thickness maps (generated based on the standardized FreeSurfer pipeline with combined T1-/T2-reconstruction [7, 8]). For this study, the high-resolution cortical thickness maps (164 k mesh) were used.

### Statistical analysis

﻿Statistical analysis of the minimally preprocessed HCP neuroimaging data was carried out with tools from the Connectome Workbench [18, 19] and MATLAB R2019b (The Mathworks, Natick, USA). First, null distributions were generated using different strategies and subsequently, these methods were validated and compared based on their specificity and sensitivity.

#### Generating null distributions

**Fig. 1:**
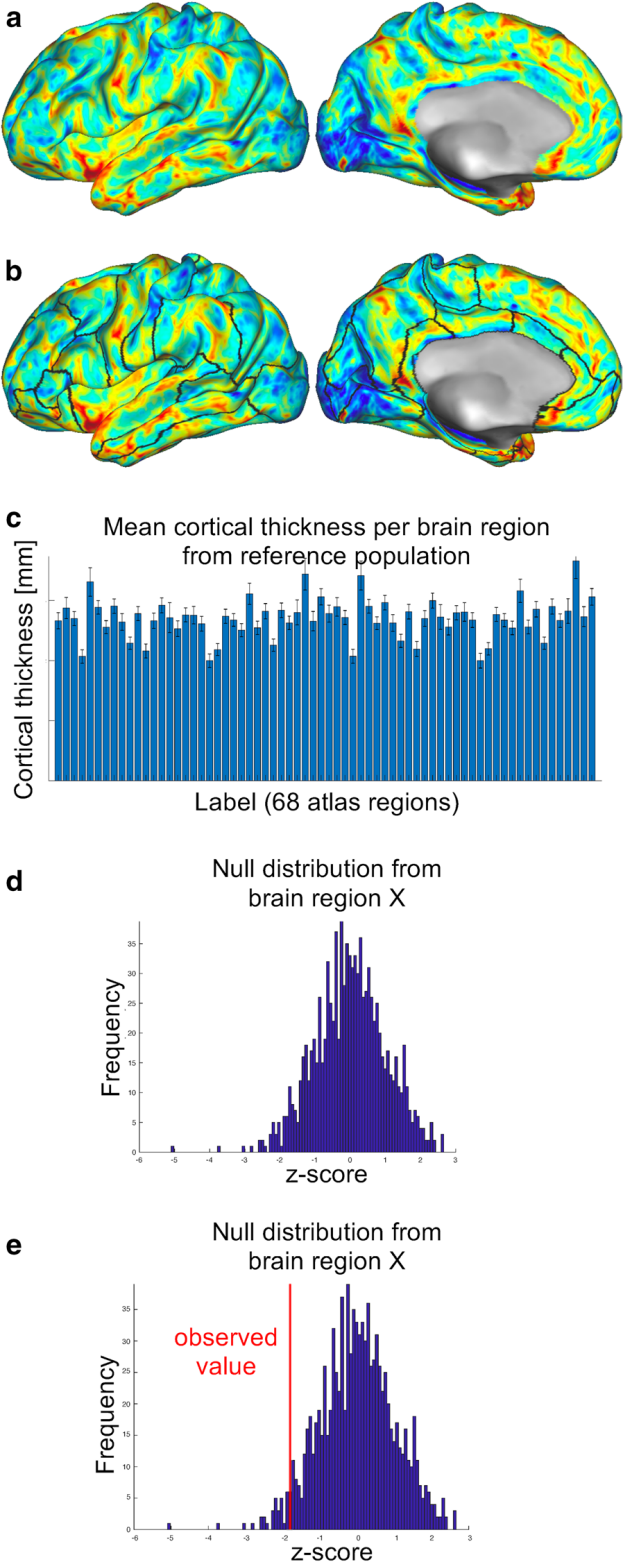
Generating a reference system for rating an individual’s cortical thickness data with respect to a control population. In methods 3 and 4, each cortical thickness map from a population sample (**a**) was divided into 68 distinct brain regions (borders are indicated as black lines in **b**). Given that the different brain regions have different means and standard deviations (**c**), this approach is biologically more plausible than generating one common reference system for all brain regions (as was tested here in methods 1 and 2). Based on these null distributions (see **d** for an example), the observed values for an individual can be rated within the control population (see red line in **e**) and statistically significant cortical thinning (i.e. atrophy) can be assessed

Different strategies to generate null distributions were compared. These can be subdivided into (a) generating one common null distribution for all data points on the cortex (referred to as “vertices” in CIFTI mesh files) and (b) generating separate null distributions for distinct brain regions (Fig. 1a, b). Note that thickness spreads nonuniformly across the human cortex [24–27] such that different brain regions show different population means (Fig. 1c). Therefore, different null distributions for distinct brain regions might increase sensitivity of detecting atrophy, which is why both approaches were compared in the present study. The two approaches were subdivided further into more and less conservative statistical corrections, such that in total, four methods were compared. Null distributions were computed using nonparametric permutation procedures for all methods [28], since they make less assumptions than parametric models and are therefore considered more robust than parametric tests [29, 30].

##### Method 1: Z-min statistic per data point

The statistically most conservative approach was based on generating one common reference distribution for all 298,261 data points of the cortical surface. First, from 1000 HCP data sets, each data set was selected iteratively (“test data set”) and standardized with respect to the remaining 999 data sets (“control data sets”). For that, z-scores were calculated for each vertex using the formula z_vertex_ = (d_vertex_ – μ_vertex_)/σ_vertex_, whereas d_vertex_ is the cortical thickness value of one vertex from the test data set, μ_vertex_ the mean value of that vertex from the control data sets and σ_vertex_ the respective standard deviation. From the resulting z-score map, only the minimum value was saved (note that the present research question specifically addresses cortical *thinning*). The result was a reference distribution consisting of 1000 z-scores. Using this distribution, each vertex of an independent validation data set can be rated separately with respect to the reference population, by z-transforming each vertex using the above formula (see section “Validation”).

##### Method 2: Z-min statistic per data point, averaged across brain regions

In method 1, a null distribution was calculated based on the most extreme values across the cortex. However, given that cortical thickness is nonuniformly distributed across the cortex physiologically [27], potential atrophy will be hard to detect in physiologically thicker brain regions. Method 2 aimed to increase the biological plausibility of the previous method. While the same null distribution was used as in method 1, in method 2, data points were summarized across anatomically distinct brain regions, defined by the Desikan–Killiany atlas [31]. This atlas subdivides the cortical surface into 68 regions based on morphologic features (“labels”, 34 on each hemisphere). For subsequent validation, statistical significance was determined for the synopsis of all vertices within each of the 68 regions, instead of for each vertex separately (see section “Validation”).

##### Method 3: Z-min statistic per brain region

In spite of the increased biological plausibility in method 2, that procedure was still based on one common null distribution from the most extreme values of the cortex. In method 3, this was corrected by calculating distinct null distributions for each of the 68 Desikan–Killiany-labels. For that, the permutation procedure described in method 1 was repeated, however now z-maps were calculated using the formula z_vertex_ = (d_vertex_ – μ_Label_)/σ_Label_, whereas z_vertex_ was the z-score for a vertex of the test data set, d_vertex_ is the observed cortical thickness value for that vertex from the test data set, μ_Label_ is the mean value of the respective *label* from the control data sets and σ_Label_ its respective standard deviation. On each iteration, the minimum z-score of all vertices composing one common label was saved, such that the result was a 68x1000 matrix, providing a null distribution for each label (Fig. 1d). With these null distributions, each brain region can be rated separately with respect to the reference population, by converting the cortical thickness data into z-scores using the formula z_Label_ = (d_Label_ – μ_Label_)/σ_Label_ (Fig. 1e).

##### Method 4: Z-score per brain region

Finally, in method 4, null distributions were generated based on *averaging across all vertices* from each brain region instead of using each label’s most extreme values, as in method 3. Mean values were calculated for each brain region of the test data set to derive null distributions. These null distributions were generated in analogy to method 3, using the formula z_Label_ = (d_Label_ – μ_Label_)/σ_Label_. Similar to method 3, also in method 4, each brain region can be rated separately with respect to the reference population, by converting the cortical thickness data into z-scores using the formula z_Label_ = (d_Label_ – μ_Label_)/σ_Label_.

#### Validation

To validate and compare the proposed methods, specificity and sensitivity were calculated. These measures were calculated for each vertex (method 1) or each label (methods 2–4) separately. For that, the 113 data sets (“validation data sets”) from the 1113 HCP data sets were used which had been spared for the generation of null distributions (see section “Subjects”). Statistical inference tests based on the null hypothesis of no atrophy for a given validation data set were carried out using the above-generated null distributions. For each vertex/label, the number of values of the null distributions that were lower than the observed cortical thickness values in a given validation data set were counted. Dividing this sum by the number of permutations (n = 1000) yielded FWER-corrected p-values (p_FWER_) [32, 33]. Vertices/labels with p_FWER_ <= 0.05 were considered to indicate lower cortical thickness values than would not be predicted by chance and therefore labeled as “atrophic”. In method 2, since data points were summarized within each label, a label was defined as “atrophic” if a certain percentage of its vertices showed p_FWER_ <= 0.05. Different percentages were tested (1%, 5%, 10%, 20%, 30%, 40%, 50%). Given that all of these thresholds yielded similarly poor results, hereafter only the results for one threshold (5%, arbitrary choice) are provided. The data for the other thresholds are provided in Additional files 1 and 2.

##### Specificity

Specificity defines the rate of true negatives, i.e. the share of patients which are correctly diagnosed as not having the condition of interest (here, “no atrophy”). The validation data set was used to calculate specificity, assuming that—given this data set was a random selection of a data set of healthy young subjects with no history of psychiatric/neurologic disorders—the validation data set can be labeled as non-atrophic. Each of the four methods was applied to all of the 113 validation data sets and specificity was defined as the percentage of vertices (method 1)/labels (methods 2, 3, 4) which were not classified as significantly atrophic. This procedure was repeated for each validation data set separately. Mean and standard deviations of the specificity calculations were determined across all 113 data sets (“cumulative specificity”).

To allow evaluation for distinct brain regions, in addition, specificity per atlas region was defined (for methods 2,3 and 4 only, since in method 1, no atlas regions were analyzed). This was done by calculating, per atlas region, the percentage of the 113 validation data sets which were not significantly classified as atrophic in that atlas region (“regional specificity”).

##### Sensitivity

**Fig. 2:**
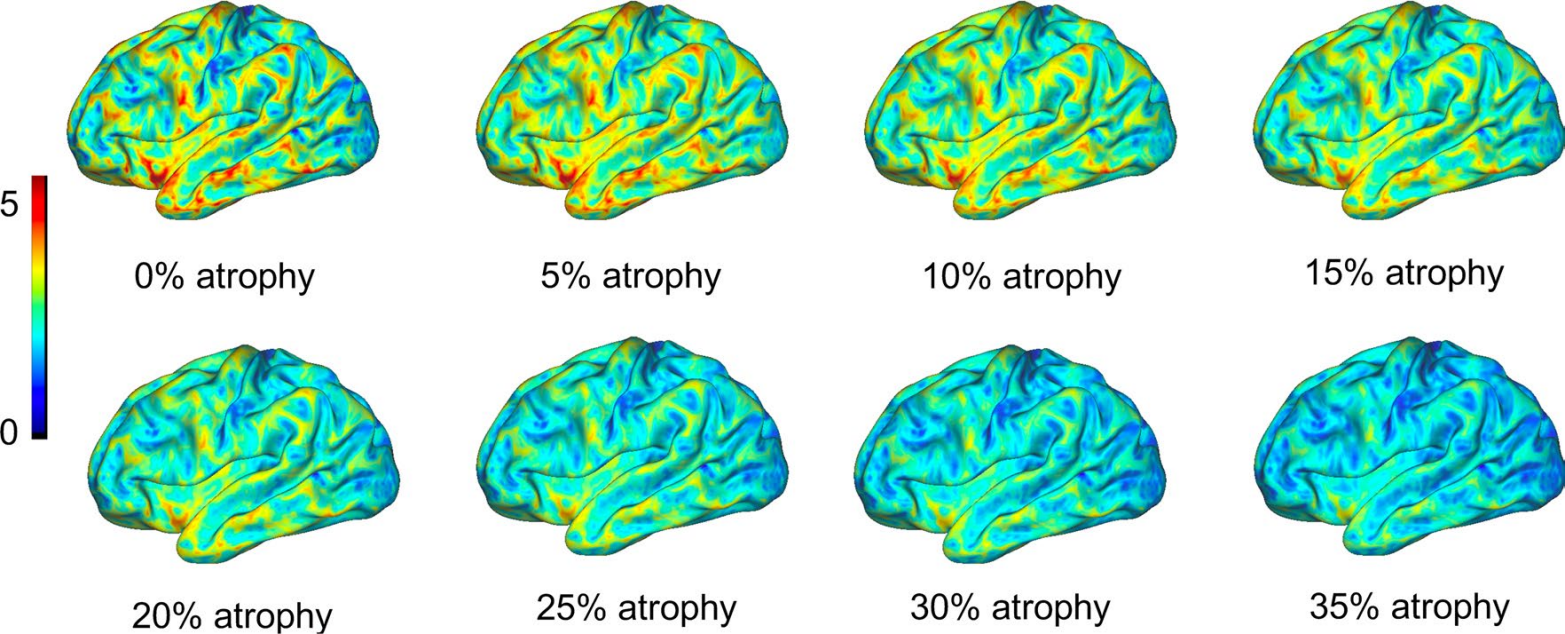
Atrophy was simulated for sensitivity calculations as follows: The original cortical thickness map from each of the subjects from the control population (“0% atrophy”) was multiplied by values ranging between 0 and 1. Multiplication by lower values indicate higher degrees of simulated atrophy. For example, multiplication by 0.9 simulates 10% atrophy, multiplication by 0.8 20% atrophy, etc. In the present study, atrophy was simulated between 1% and 100% in steps of one percentage points. This allows to assess sensitivity by the degree of simulated atrophy. In this Figure, coloring indicates cortical thickness in millimeters

Sensitivity defines the rate of true positives, i.e. the share of patients which are correctly diagnosed as having the condition of interest (here, “atrophy”). Given no true atrophy was assumed in the validation data sets, atrophy was simulated: Different degrees of atrophy were simulated as follows (Fig. 2): The original cortical thickness data (each vertex) was multiplied by a number between 0 and 1 (e.g. multiplication by 0.9 represents simulated atrophy of 10%, etc.). For each of the 113 validation data sets, atrophy was simulated from 1% to 100% in steps of 1 percentage points (p.p.). Then, each of the four methods was applied to all of the simulated data sets. For each method and degree of atrophy, sensitivity was calculated separately. Cumulative sensitivity was defined as the percentage of vertices (method 1) or labels (methods 2, 3, 4) which were classified as significantly atrophic, summarized across all 113 data sets (“cumulative sensitivity”). Sensitivity across methods was compared using the degree of atrophy required to achieve cumulative sensitivity of 80% (“cumulative sensitivity threshold”). Note that less sensitive methods will require more pronounced atrophy, therefore a higher cumulative sensitivity threshold, in order to detect atrophy.

To allow evaluation for distinct brain regions, additionally, sensitivity per atlas region was defined for each degree of atrophy (for methods 2,3 and 4 only, since in method 1, no atlas regions were analyzed). This was done by calculating, per atlas region, the percentage of the 113 validation data sets which were significantly classified as atrophic in that atlas region (“regional sensitivity”).

Note that although cortical thickness was simulated at consistent rates throughout the cortex (which is not how cortical thinning occurs in aging or pathology [10, 15, 16]), evaluation was performed for each vertex/label independently. Therefore, the proposed methods are fit to analyze also diffuse patterns of cortical thinning.

## Results

### Specificity

**Fig. 3:**
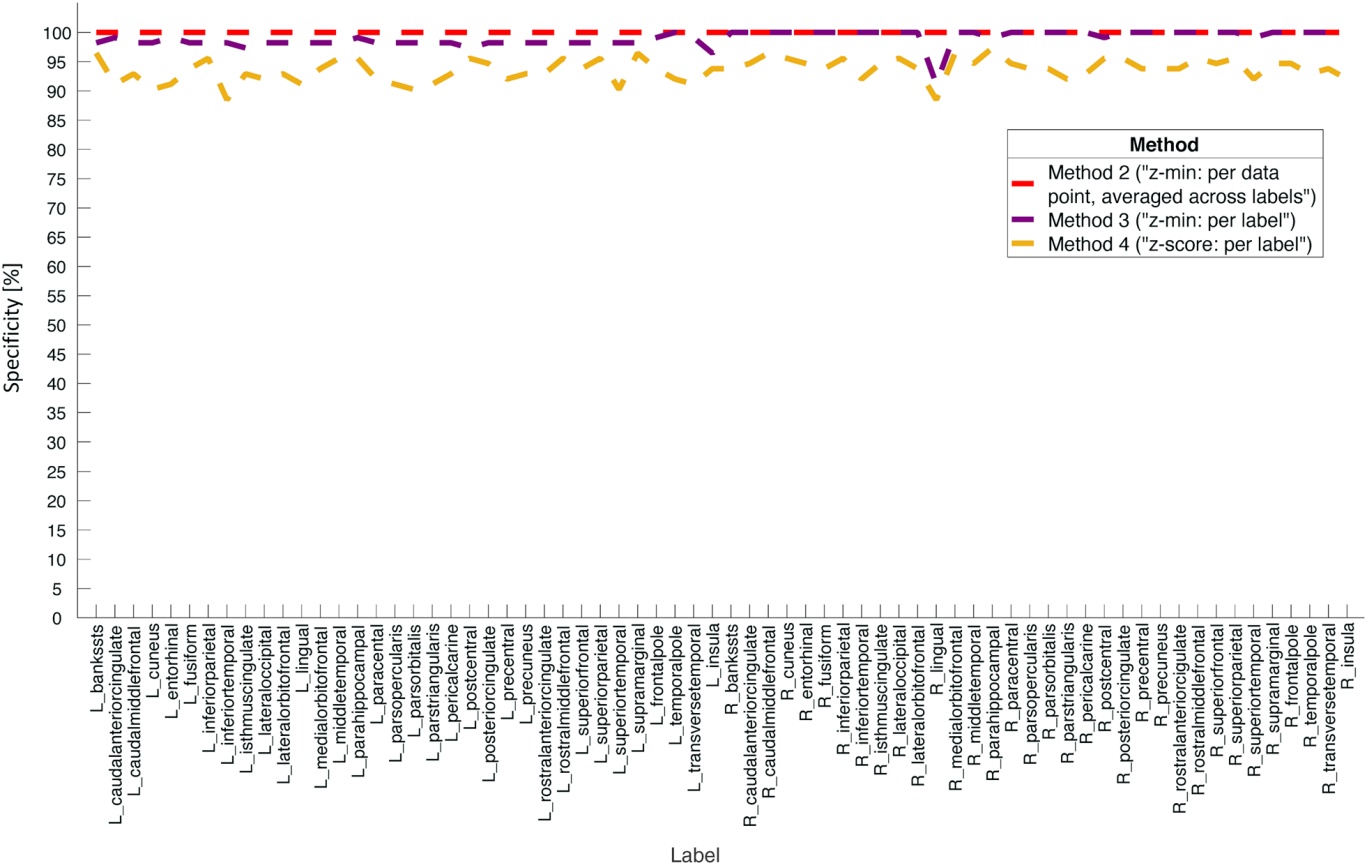
Comparison of regional specificity profiles between methods 2–4. The statistically most conservative approach (method 2, “z-min: per data point, averaged across labels”, red dashed line) yielded ideal specificity for all brain regions, i.e. it correctly assigns “no atrophy” in 100% of cases. The less conservative method 3 (“z-min: per label”, purple dashed line) also showed specificity of 100% for many brain regions, but had some drops, e.g. for the right lingual gyrus. The most liberal approach, method 4 (“z-score: per label”, golden dashed line) yielded lower specificity for all brain regions. Note that method 1 (“z-min: per data point”) is not shown here because it does not allow for labelwise assessment. See also Table 1 for the cumulative specificity values for each method

Table 1 summarizes the cumulative specificity calculations for each method. Methods 1 and 2 showed ideal specificity (100%, ± 0 p.p.), such that these methods classified no vertex (method 1)/label (method 2) as significantly atrophic. Method 3 had a mean specificity of 98.9% (± 1.3 p.p.), and method 4 was less specific with a mean of 93.6% (± 2.0 p.p.). Figure 3 shows the regional specificity profiles evaluated across all 68 atlas regions. While the most specific method (method 2, red dashed line) yielded 100% specificity for each label, method 3 showed relatively constant specificity across brain regions except for a slight drop for the right lingual gyrus. Method 3 showed specificity of 100% for almost all labels on the right hemisphere (notice however a slight drop for the right lingual gyrus), while the values were slightly lower for the labels on the left hemisphere. Finally, method 4 (golden dashed line) showed notably lower values throughout all labels as compared to methods 2 and 3.

**Table 1:** Cumulative specificity calculations for the four tested methods

	Method 1 (“z-min: per data point”)	Method 2 (“z-min: per data point, averaged across labels”)	Method 3 (“z-min: per label”)	Method 4 (“z-score: per label”)
Mean specificity (across subjects)	100%	100%	98.9%	93.6%
Standard deviation (across subjects)	0.0 p.p.	0.0 p.p.	1.3 p.p.	2.0 p.p.

p.p.: percentage point(s)

### Sensitivity

**Fig. 4:**
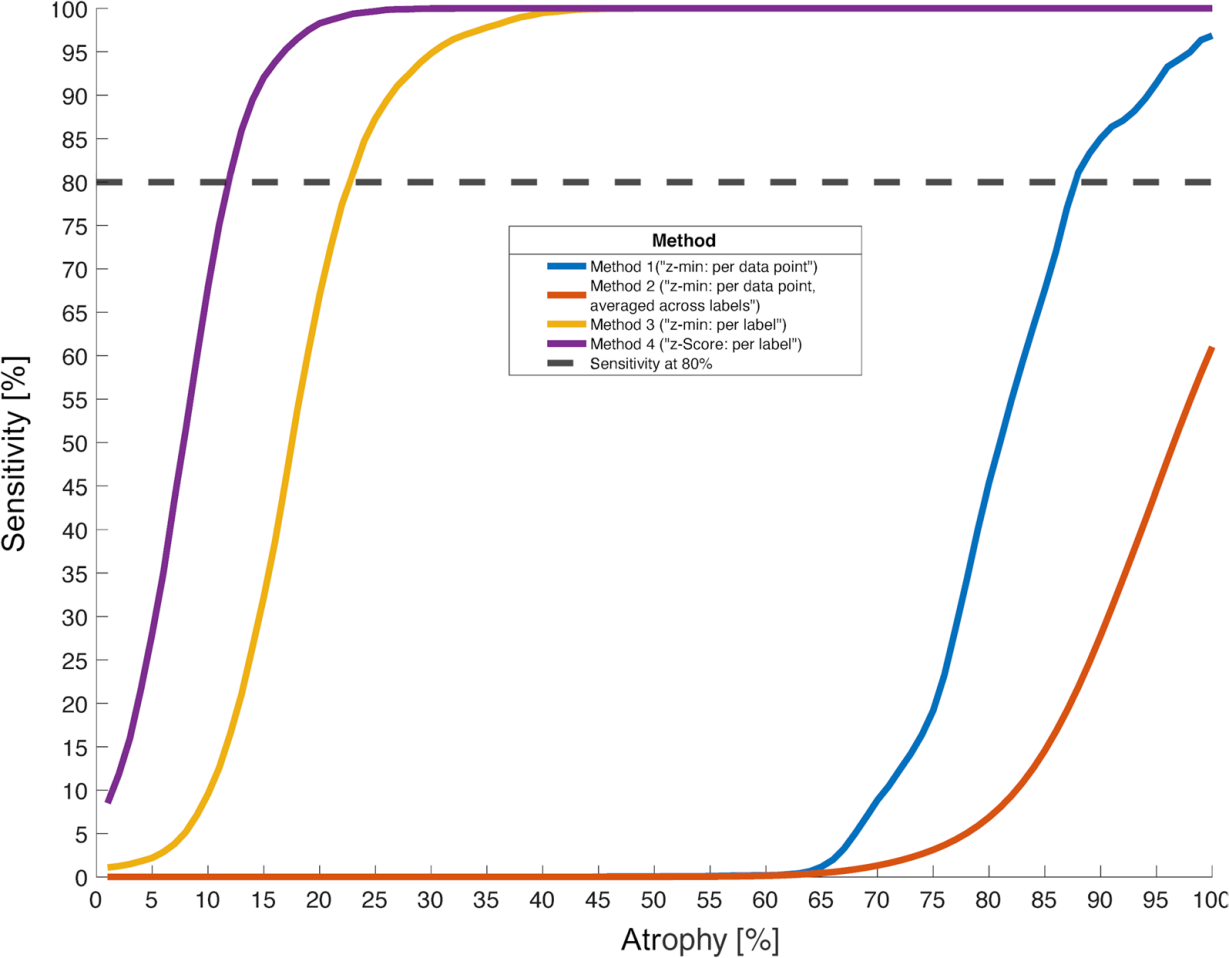
Cumulative sensitivity relative to the degree of simulated atrophy (across vertices/brain regions), comparison between the four tested methods. All methods detected atrophy more sensitive for more pronounced degrees of atrophy. However, the degree of atrophy the methods required to reach a given level of sensitivity differed. For example, in the current simulation, in order to detect atrophy in 80% of cases (black horizontal dashed line), method 4 (“z-score: per label”, purple line) required only 12% atrophy, method 3 (“z-min: per label”, golden line) 23%, method 2 (“z-min: per data point, averaged across labels”, blue line) 88%, while method 1 (“z-min: per data point”, red line) failed to detect atrophy in 80% of cases even for the highest possible degree of atrophy (100%). Compare also Table 2 for a summary of these results

Figure 4 illustrates the cumulative sensitivity profiles for each method relative to the degree of simulated atrophy. The horizontal dashed line denotes sensitivity at 80% (cumulative sensitivity threshold), which was used to compare the different methods. Table 2 summarizes these results: Method 1 (red line) was extremely unsensitive, such that not even for the highest possible degree of atrophy (literally no brain) did this method detect atrophy in 80% of cases (i.e. cumulative sensitivity threshold not reached). Method 2 (blue line) yielded a cumulative sensitivity threshold for 88% simulated atrophy when a label was considered atrophic if 5% of its vertices had p_FWER_ < 0.05 (see “Methods”). Other tested thresholds for method 2 comprised 1% (cumulative sensitivity threshold for 84% simulated atrophy), 10% (90% atrophy), 20% (94% atrophy), 30% (98% atrophy), 40%/50% (did not reach 80% sensitivity for any degree of simulated atrophy, see Additional file 1: Fig. S1 and Additional file 2: Table S1). Method 3 (yellow line) was clearly superior (cumulative sensitivity threshold 23% simulated atrophy), and for method 4 an even lower value (12% simulated atrophy) was observed.

**Table 2:** Cumulative sensitivity thresholds for the four tested methods

	Method 1 (“z-min: per data point”)	Method 2 (“z-min: per data point, averaged across labels”)	Method 3 (“z-min: per label”)	Method 4 (“z-score: per label”)
Degree of atrophy required for detection of atrophy in 80% of cases (cumulative sensitivity threshold)*	Not available	88%	23%	12%

* Note that lower values of atrophy suggest more sensitive methods, since they detect less pronounced atrophy

**Fig. 5:**
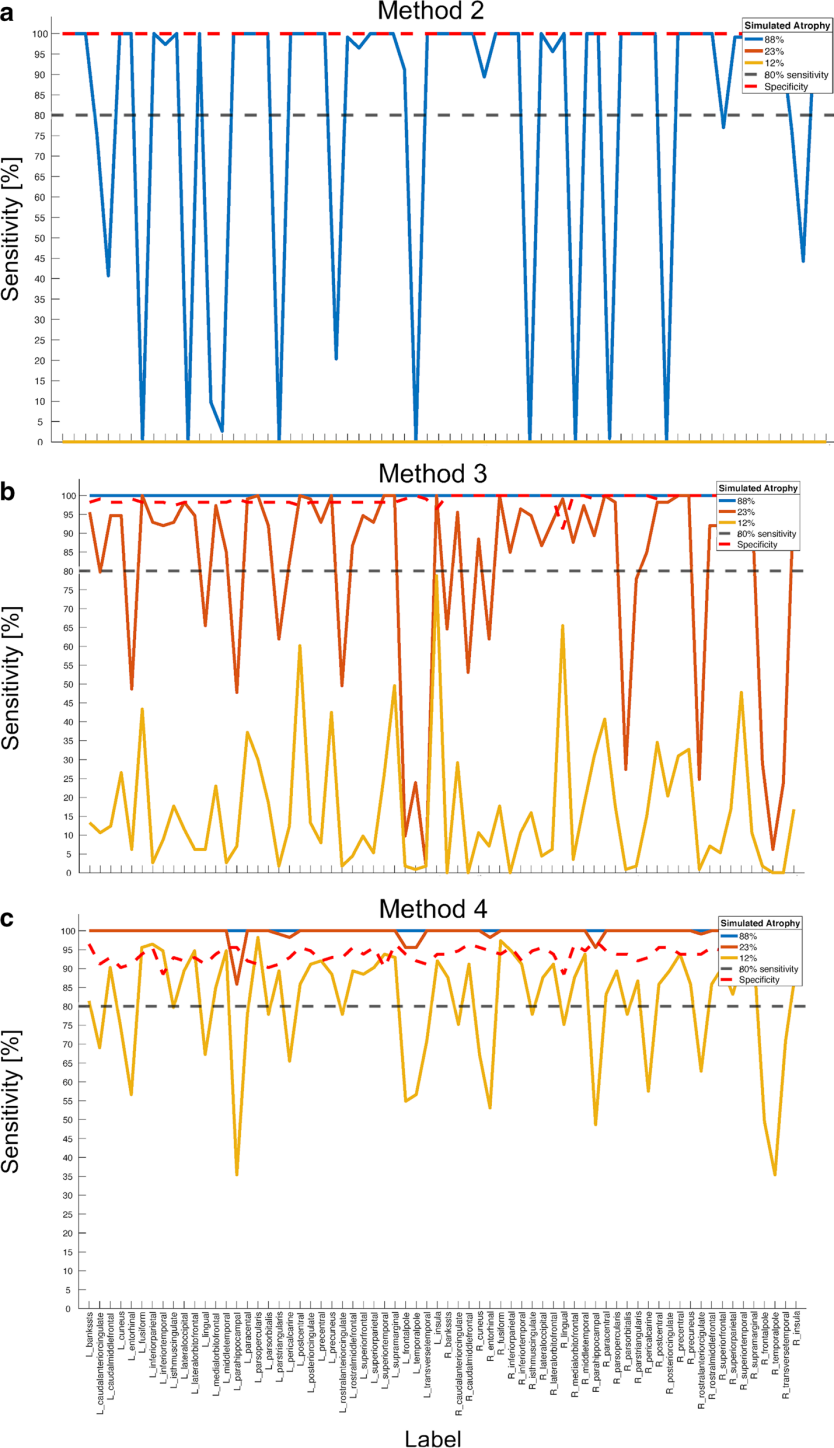
Regional sensitivity (per brain region) for each region’s cumulative sensitivity threshold (i.e. the degree of atrophy each method required to detect atrophy in 80% of cases) for method 2 (**a**, “z-min: per data point, averaged across labels”, method 3 (**b**, “z-min: per label”) and method 4 (**c**, “z-score: per label”). The cumulative sensitivity threshold for method 2 was 88% atrophy (blue lines), for method 3 23% atrophy (red lines) and for method 4 12% atrophy (golden lines). The 80% sensitivity line is indicated by the gray dashed lines in each panel. In addition, regional specificity is plotted for each method (red dashed lines, compare also Fig. 3). All methods detected atrophy more sensitively for more pronounced degrees of atrophy

Figure 5 shows the results of the regional sensitivity determination for methods 2 (Fig. 5a), 3 (Fig. 5b) and 4 (Fig. 5c). To compare the methods, the regional sensitivity profiles are plotted for each method’s cumulative sensitivity threshold (i.e. 88% atrophy for method 2: blue lines, 23% atrophy for method 3: red lines, 12% atrophy for method 4: golden lines). To enhance orientation, 80% sensitivity is indicated with a gray dashed line in Fig. 5a–c. Additionally, regional specificity for each method is plotted (red dashed lines).

Figure 5a illustrates poor sensitivity of method 2, given it reaches sensitivity of > 0% for none of the cumulative sensitivity thresholds of the other methods. Additionally, the regional sensitivity profile for its own cumulative sensitivity threshold (88% simulated atrophy) shows strong variations across labels. Method 3 (Fig. 5b) is clearly superior: while the variations for its own cumulative sensitivity threshold (23% simulated atrophy) are less pronounced as compared to method 2, it yields perfect (i.e. 100%) sensitivity for the cumulative sensitivity threshold of method 2. However, no region reaches 80% sensitivity for the cumulative sensitivity threshold of method 3. Finally, method 4 (Fig. 5c) is the most sensitive of the tested methods. It yields almost perfect regional sensitivity for the cumulative sensitivity thresholds of methods 2 and 3, and the regional sensitivity profile for its own cumulative sensitivity threshold (12% simulated atrophy) shows less variations than the other methods. Note however the relatively low specificity (red dashed line) of this method as compared to the others.

Nevertheless, it is evident from Fig. 5 that there are regional variations for the cumulative sensitivity thresholds for each method. Additional file 3: Table S2 lists the labels which show less regional sensitivity than 80% for each method and their respective cumulative sensitivity threshold. For example, for method 3, among the brain regions that yielded least sensitivity for that method’s cumulative sensitivity threshold (23% atrophy) are, on the left hemisphere, parahippocampal gyrus (49.56% sensitivity), temporal pole (23.89% sensitivity), frontal pole (9.73% sensitivity), temporal pole (23.89% sensitivity) and transverse temporal gyrus (1.77% sensitivity), and on the right hemisphere, pars orbitalis (27.43% sensitivity), rostral anterior cingulate (24.78% sensitivity), frontal pole (29.20% sensitivity), temporal pole (6.19% sensitivity) and transverse temporal gyrus (23.89% sensitivity).

## Discussion

The goal of this study was to develop a method which allows to rate a single patient’s cortical thickness data and identify atrophy sensitively and specifically with respect to a control population. This study was motivated by the many previous reports which have found pronounced associations of cortical thinning with the diagnosis/progression of diverse neurological and psychiatric conditions. In addition, given that different pathologies present with different patterns of cortical thinning, another goal was to allow the evaluation of cortical thinning for distinct brain regions. To provide such a resource, a reference system was developed by generating population-based distributions of expected cortical thickness data, both for the entire cortex as well as for distinct brain regions. 1000 data sets from young and healthy participants were used to generate expected population null distributions using a permutation procedure. To assess statistically significant cortical thinning (i.e. atrophy), different methods were tested and compared using sensitivity and specificity calculations for the entire cortex (“cumulative”) as well as for distinct brain regions (“regional”), calculated from 113 additional subjects. The statistically most stringent methods were based on one common null distribution for all brain regions, which showed ideal specificity but poor sensitivity. Other methods were based on distinct null distributions for different brain regions, which increased sensitivity but decreased specificity. However, when generating distinct null distributions for different brain regions based on the most extreme values within each label (method 3), the drop in cumulative specificity was only very subtle (98.9%), while cumulative sensitivity could still be detected at 80% for 23% simulated atrophy. Variations of regional differences were observed for some brain regions, but decreased for more pronounced degrees of atrophy.

These results emphasize that in order to sensitively detect cortical atrophy for individual patients, it is reasonable to create different null distributions for distinct brain regions. Cortical thickness is not spread uniformly across the cortex [34], such that for example neurite density is higher for motor regions as compared to regions associated with higher cognitive functions [27]. Therefore, a single reference distribution to rate any cortex region is biologically implausible and will result in decreases of sensitivity, which was shown here in methods 1 and 2. Furthermore, with this approach, sensitivity is relatively constant for different brain regions, although regional variations are observed (Fig. 5b).

One drawback of working with several null distributions for different brain regions as opposed to a common one is that specificity decreases, which was shown in methods 3 and 4. In method 3, a strategy was suggested to minimize this loss in specificity while maintaining a high level of sensitivity: The idea of method 3 was to generate null distributions for different brain regions based on the (minimally) most extreme values within each brain region across a control population, instead of working with averages across brain regions. With this strategy, atrophy could be detected in 80% of cases when the cortex was roughly three quarters of its original thickness. However, in cases where the clinician wishes to detect atrophy more sensitively, method 4 might be preferred—there, null distributions were generated from population *averages* (rather than from their most extreme values). In this study, that method could detect atrophy in 80% of cases already when the cortex was thinned by a factor of only 12% (also here, regional variations were observed, see Fig. 5c). However, that approach would imply risking to detect false positives, given its lower specificity. Depending on the situation, the clinician can flexibly choose between more sensitivity or more specificity.

**Fig. 6:**
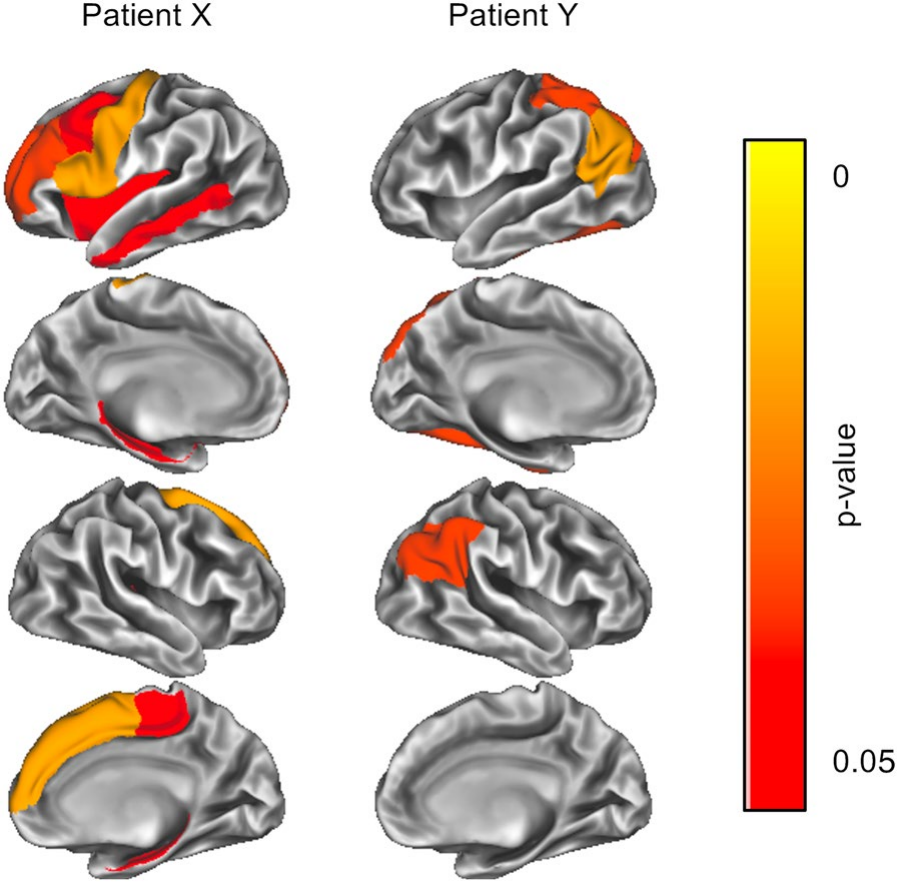
Exemplary result of analyzing a T1-weighted MRI data set with the current methods. For patient X, cortical thinning was simulated in frontal regions, for patient Y in more posterior regions. Method 3 was used to analyze the data. The emerging p-map indicates where cortical thinning likely occurs in each patient. Using the method proposed in this text, such maps can be created easily and are therefore simple to implement into clinical practice

One limitation of the suggested reference system is that it was generated from a relatively homogenous control population of healthy young adults. However, cortical thickness declines even in physiological aging, such that the comparison of an elderly individual to that reference group will result in more pronounced atrophy detection, which would not necessarily have to be pathologic [10, 15]. Nevertheless, given that the regions that exhibit cortical thinning differ in physiological and pathological aging (for example, atrophy of brain regions such as the precuneus and the inferior temporal region can be indicative of early signs of dementia [35]), it is still possible to detect such potential pathologic signatures using the method proposed here. This is possible because the reference system suggested herein was generated and evaluated for different brain regions separately. This allows to rate different brain regions independently, such that different atrophy patterns can be identified. Figure 6 illustrates this: For patient X, atrophy was simulated in frontal areas, for patient Y in more posterior regions. Using method 3, the resulting p-map indicates where cortical thinning occurred for that patient. Such maps can be generated easily with a given patient’s T1-weighted MRI using the procedure proposed here, and are therefore easy to implement into clinical practice.

The atlas used in this work was the Desikan–Killiany atlas, a brain atlas defined by morphologic features of the cortex and therefore surface-based. This is an important feature because cortical thinning is modified by genetic components [15, 16], and such genetic patterns yield high resemblance to surface-based features [36]. Additionally, patterns of genetic overlap seem to be coarse-grained across the human cortex (current optimal solutions suggest between 9 and 12 labels per hemisphere [16, 36]), such that the Desikan–Killiany atlas (34 labels per hemisphere) allows a more fine-grained resolution than proposed by genetic commonalities. However, especially in early pathology, cortical thinning may be more localized, such that future work should investigate the benefit of using a more fine-grained atlas for such cases. Furthermore, a more fine-grained atlas might also help to enhance regional sensitivity of those brain regions which showed poor sensitivity with the Desikan–Killiany altas (such as the left frontal pole as well as the left and right transverse temporal gyri). The evaluation of these regions with the current method and atlas should be made with caution given their lower sensitivity.

Finally, the current reference system allows to progress-monitor an individual’s condition: given the composition of the reference standard does not change, any potential changes between two measurement time points can be more likely attributed to changes in the individual. Finally, it should be emphasized that atrophy was only simulated in this study, and it is subject to future work to validate the present simulations with real data. It will also be necessary to show that the system is applicable to data acquired from different types of MR scanners and sequence parameters (here, data from a 3 Tesla MR scanner with optimized parameters for T1-weighted imaging were analyzed).

## Conclusions

Taken together, the here suggested reference system can be used for sensitive and specific detection of cortical atrophy for distinct brain regions (defined by the Desikan–Killiany atlas) for age groups comparable to the reference population (22–40 years), which allows to detect differential patterns of cortical thinning. However, some brain regions are detected less sensitively such that those regions should be evaluated with care. The method should therefore be further validated with data from different pathologies and using different atlases. Although distinct reference systems for different age groups will further help to establish this method in clinical practice, the current method already allows to rate elderly individuals, however these cases should be treated with caution given the risk of detecting false positives due to effects of physiological aging. However, progress-monitoring of elderly individuals is possible with the current system if the individual is compared to its own ranking within the control population for each measurement time point. Therefore, the tool proposed in this work represents a first step of the translation of cortical thickness measures into clinical practice.

## Supplementary information

**Additional file 1: Figure S1.** Cumulative sensitivity relative to the degree of simulated atrophy (across vertices/brain regions), comparison between the four tested methods and different thresholds for method 2. In method 2, a label was defined “atrophic” if a certain percentage of its vertices yielded p_FWER_ <= 0.05. Here, the results for thresholds 1%, 5% (which is shown in the main text), 10%, 20%, 30%, 40% and 50% are displayed

**Additional file 2: Table S1.** Cumulative sensitivity calculations for different thresholds for method 2 (in method 2, a label was defined “atrophic” if a certain percentage of each label’s vertices yielded p_FWER_ <= 0.05).

**Additional file 3: Table S2**. For methods 2,3 and 4, cumulative sensitivity was defined based on the degree of simulated atrophy a method required to sensitively detect 80% (method 2: 88% simulated atrophy, method 3: 23% simulated atrophy, method 4: 12% simulated atrophy). However, regional sensitivity varied for that degree of atrophy. This table indicates which labels showed < 80% sensitivity for each method’s “crucial” degree of atrophy, along with the regional sensitivity detected for that degree of atrophy.

## Data Availability

All data used in this study are freely and openly available for scientific interrogations from the Human Connectome Project. Researchers can access them online at https://db.humanconnectome.org/app/template/Login.vm;jsessionid=A3E03522D3DEC91B2D2A09FB80CCE6CF.

## Abbreviations

BW: Bandwidth
FA: Flip angle
Fig: Figure
FOV: Field of view
GM: Gray matter
HCP: Human Connectome Project
MRI: Magnetic resonance imaging
p.p.: Percentage point(s)
TE: Echo time
TR: Repetition time
VR: Voxel resolution
