# Supplementary figures and images for: Towards individualized cortical thickness assessment for clinical routine
Source: J Transl Med. 2020 Apr 3;18:151. doi: 10.1186/s12967-020-02317-9 (PMC7118882; doi:10.1186/s12967-020-02317-9)

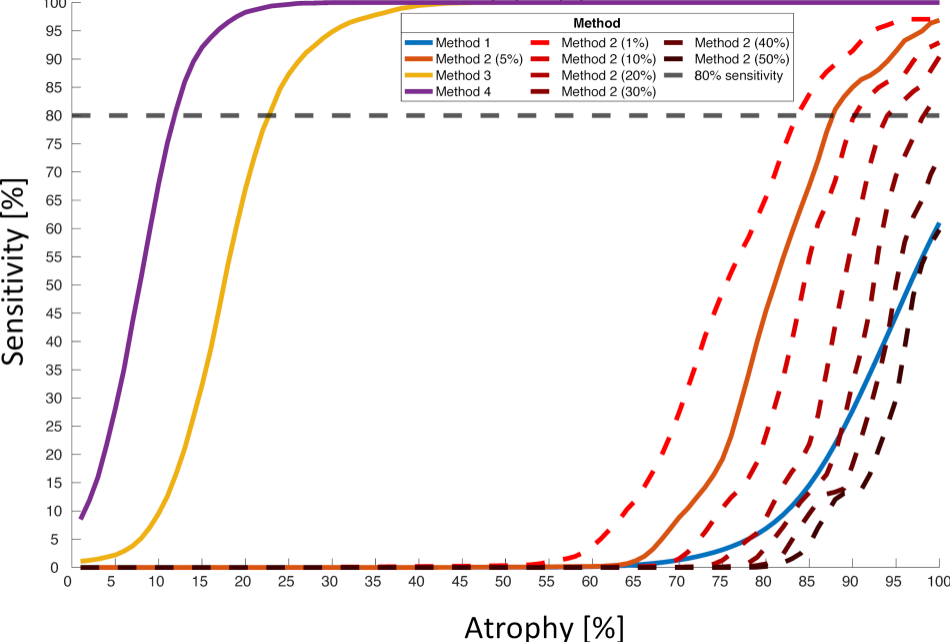

Supplement: Supplementary file 1 — Additional file 1: Figure S1. Cumulative sensitivity relative to the degree of simulated atrophy (across vertices/brain regions), comparison between the four tested methods and different thresholds for method 2. In method 2, a label was defined “atrophic” if a certain percentage of its vertices yielded pFWER <= 0.05. Here, the results for thresholds 1%, 5% (which is shown in the main text), 10%, 20%, 30%, 40% and 50% are displayed [file 12967_2020_2317_MOESM1_ESM.pdf]
