# Supplementary material for: Towards individualized cortical thickness assessment for clinical routine
Source: J Transl Med. 2020 Apr 3;18:151. doi: 10.1186/s12967-020-02317-9 (PMC7118882; doi:10.1186/s12967-020-02317-9)
Supplement: Supplementary file 2 — Additional file 2: Table S1. Cumulative sensitivity calculations for different thresholds for method 2 (in method 2, a label was defined “atrophic” if a certain percentage of each label’s vertices yielded pFWER <= 0.05). [file 12967_2020_2317_MOESM2_ESM.docx]

## Additional file 2

**Table S1.** Cumulative sensitivity calculations for different thresholds for method 2 (in method 2, a label was defined “atrophic” if a certain percentage of each label’s vertices yielded p_FWER_ < 0.05).

|  | **1%** | **5%** | **10%** | **20%** | **30%** | **40%** | **50%** |
| --- | --- | --- | --- | --- | --- | --- | --- |
| **Degree of atrophy required for detection of atrophy in 80% of cases (sensitivity**)* | 84% | 88 % | 90% | 94% | 98% | n.a. | n.a. |

* Note that lower values of atrophy suggest more sensitive methods, since they detect less pronounced atrophy.

Abbreviations: n.a. = not available
