# Supplementary material for: Towards individualized cortical thickness assessment for clinical routine
Source: J Transl Med. 2020 Apr 3;18:151. doi: 10.1186/s12967-020-02317-9 (PMC7118882; doi:10.1186/s12967-020-02317-9)
Supplement: Supplementary file 3 — Additional file 3: Table S2. For methods 2,3 and 4, cumulative sensitivity was defined based on the degree of simulated atrophy a method required to sensitively detect 80% (method 2: 88% simulated atrophy, method 3: 23% simulated atrophy, method 4: 12% simulated atrophy). However, regional sensitivity varied for that degree of atrophy. This table indicates which labels showed < 80% sensitivity for each method’s “crucial” degree of atrophy, along with the regional sensitivity detected for that degree of atrophy. [file 12967_2020_2317_MOESM3_ESM.docx]

## Additional file 3

**Table S2**. For methods 2,3 and 4, cumulative sensitivity was defined based on the degree of simulated atrophy a method required to sensitively detect 80% (method 2: 88%, method 3: 23%, method 4: 12%). However, regional sensitivity varied for that degree of atrophy. This table indicates which labels showed < 80% sensitivity for each method’s “crucial” degree of atrophy, along with the regional sensitivity detected for that degree of atrophy.

| Method 2 (88 % simulated atrophy) | | Method 3 (23% simulated atrophy) | | Method 4 (12% simulated atrophy) | |
| --- | --- | --- | --- | --- | --- |
| Labelname | Regional sensitivity | Labelname | Regional sensitivity | Labelname | Regional sensitivity |
| L_cuneus | 75.22% | L_caudalanteriorcingulate | 0.7965 | L_caudalanteriorcingulate | 0.6903 |
| L_entorhinal | 40.71% | L_entorhinal | 0.4867 | L_cuneus | 0.7434 |
| L_inferiortemporal | 0.00% | L_lingual | 0.6549 | L_entorhinal | 0.5664 |
| L_lingual | 0.00% | L_parahippocampal | 0.4779 | L_isthmuscingulate | 0.7965 |
| L_middletemporal | 9.73% | L_parstriangularis | 0.6195 | L_lingual | 0.6726 |
| L_parahippocampal | 2.65% | L_rostralanteriorcingulate | 0.4956 | L_parahippocampal | 0.3540 |
| L_pericalcarine | 0.00% | L_frontalpole | 0.0973 | L_paracental | 0.7788 |
| L_rostralanteriorcingulate | 20.35% | L_temporalpole | 0.2389 | L_parsorbitalis | 0.7788 |
| L_temporalpole | 0.00% | L_transversetemporal | 0.0177 | L_pericalcarine | 0.6549 |
| R_inferiortemporal | 0.00% | R_bankssts | 0.6460 | L_rostralanteriorcingulate | 0.7788 |
| R_lingual | 0.00% | R_caudalmiddlefrontal | 0.5310 | L_frontalpole | 0.5487 |
| R_parahippocampal | 0.88% | R_entorhinal | 0.6195 | L_temporalpole | 0.5664 |
| R_pericalcarine | 0.00% | R_parsorbitalis | 0.2743 | L_transversetemporal | 0.7080 |
| R_rostralanteriorcigulate | 76.99% | R_parstriangularis | 0.7788 | R_caudalanteriorcingulate | 0.7522 |
| R_frontalpole | 76.11% | R_rostralanteriorcigulate | 0.2478 | R_cuneus | 0.6726 |
| R_temporalpole | 44.25% | R_frontalpole | 0.2920 | R_entorhinal' | 0.5310 |
|  |  | R_temporalpole | 0.0619 | R_isthmuscingulate | 0.7788 |
|  |  | R_transversetemporal | 0.2389 | R_lingual | 0.7522 |
|  |  |  |  | R_parahippocampal' | 0.4867 |
|  |  |  |  | R_parsorbitalis | 0.7788 |
|  |  |  |  | R_pericalcarine | 0.5752 |
|  |  |  |  | R_rostralanteriorcigulate | 0.6283 |
|  |  |  |  | R_frontalpole | 0.4956 |
|  |  |  |  | R_temporalpole | 0.3540 |
|  |  |  |  | R_transversetemporal | 0.7080 |

Abbreviations: R = right, L = left
